# Expression Profiles of the Progesterone Receptor, Cyclooxygenase-2, Growth Differentiation Factor 9, and Bone Morphogenetic Protein 15 Transcripts in the Canine Oviducts during the Oestrous Cycle

**DOI:** 10.3390/ani11020454

**Published:** 2021-02-09

**Authors:** Jaime Palomino, Javiera Flores, Georges Ramirez, Victor H. Parraguez, Monica De los Reyes

**Affiliations:** 1 Laboratory of Animal Reproduction, Department of Animal Production, Faculty of Veterinary Sciences, University of Chile, Santiago 8820000, Chile; jpalomin@veterinaria.uchile.cl (J.P.); javiera.flores.j@ug.uchile.cl (J.F.); georgimaster@hotmail.com (G.R.); 2 Laboratory of Animal Physiology, Department of Biological Sciences, Faculty of Veterinary Sciences, University of Chile, Santiago 8820000, Chile; vparragu@uchile.cl

**Keywords:** dog, gene expression, oviductal cells, anoestrus, proestrus, oestrus, dioestrus, progesterone, oestrous cycle regulation

## Abstract

**Simple Summary:**

The oestrous cycle in canines is specifically more extended than that in other mammals. This implies that the oocytes do not reach maturity within the ovarian follicle but undergo final maturation in the oviducts. Besides oocyte maturation, the oviduct provides the necessary milieu for fertilization and preimplantation embryonic development. Consequently, the oviductal environment presumably changes in the postovulatory period and throughout the entire reproductive cycle to provide a suitable condition for supporting different functions. In this study, we evaluated the gene expression of different genes associated with oocyte-embryo development, such as progesterone receptor, cyclooxygenase-2, growth differentiation factor 9, and bone morphogenetic protein 15 in the canine oviductal cells at different phases of the oestrous cycle. Using quantitative PCR (qPCR) analysis in bitch oviductal cells, this study revealed the ovarian cycle’s influence on the oviductal essential transcripts in the bitch. It also assessed the influence of the ovulated cumulus-oocytes complexes on the expression of *GDF-9* and *BMP-15* genes. Thus, the oestrous-cycle-dependent gene expression pattern of *PR*, *COX-2*, *GDF-9*, *BMP-15* in the canine oviduct was found to execute the oviductal cell interactions necessary for the development and function of the canine reproductive tract.

**Abstract:**

The gene expression in the canine oviduct, where oocyte maturation, fertilization, and early embryonic development occur, is still elusive. This study determined the oviductal expression of (*PR*), cyclooxygenase-2 *(COX-2*), growth differentiation factor 9 (*GDF-9*), and bone morphogenetic protein 15 (*BMP-15*) during the canine oestrous cycle. Samples were collected from bitches at anoestrus (9), proestrus (7), oestrus (8), and dioestrus (11), after routine ovariohysterectomy and the ovarian surface structures and plasma progesterone concentration evaluated the physiological status of each donor. The oviductal cells were isolated and pooled. Total RNA was isolated, and gene expression was assessed by qPCR followed by analysis using the *t*-test and ANOVA. The *PR* mRNA increased (*P* < 0.05) from the anoestrus to dioestrus with the plasma progesterone concentration (r = 0.8). *COX-2* mRNA expression was low in the anoestrus and proestrus, and negligible in the oestrus, while it was around 10-fold higher (*P* < 0.05) in the dioestrus. The *GDF-9* mRNA was expressed during all phases of the oestrous cycle and was most abundant (*P* < 0.05) during oestrus phase. The *BMP-15* mRNA decreased (*P* < 0.05) in the anoestrus and proestrus phases. Thus, the transcripts were differentially expressed in a stage-dependent manner, suggesting the importance of oestrous cycle regulation for successful reproduction in dogs.

## 1. Introduction

One of the most intriguing characteristics of canine reproductive physiology is the long oocyte maturation process, which prolongs the presence of the oocyte at the oviductal level. Although the oviducts play a decisive role for final oocyte maturation and also is an important environment for gamete interaction, fertilization, and early embryonic development, the oviduct micro-environment is still poorly understood in canines.

Several agents such as the ovarian hormones [1,2,3], prostaglandins [4,5], and growth factors [6] are known to influence oviductal functions in several species [7].

Ovarian steroids, including progesterone, affect the oviduct by regulating secretory functions in the lumen [3,8], which in turns affect the oocyte final maturation and embryo growth. Maturation of the canine oocytes occurs in a progesterone dominant environment, because during the oestrus stage the follicles luteinize, changing the secretion to progesterone [9]; therefore, after ovulation progesterone- can be considered an important mediator of the oviductal microenvironment that can facilitate oocyte maturation, fertilization, and early embryonic development. 

The major physiological effect of progesterone is mediated by the nuclear progesterone receptor (PR) [10,11]. In mammals, the *PR* gene gives rise to two functionally distinct protein isoforms, PR-A and PR-B [12], and both are expressed in the oviduct of different mammals [13,14], including canines [15]. From genomic approaches, *PR* has been identified as an important regulator of gene transcription [16]. Many physiological effects of progesterone are mediated by cyclooxygenase-2 as an inducible and rate-limiting enzyme in the synthesis of prostaglandins, which convert arachidonic acid into prostaglandin [17,18]. The in vitro *COX-2* mRNA expression is stimulated after progesterone treatment [5,19] and, prostaglandin-2 (PGE2) function as a luteotropic factor in dogs [20], promoting the premature luteinization associated with the high progesterone concentration at the follicular and peripheral levels [21]. Different reports in other species reinforce that COX-2-derived PGE2 is at least one of the key players in regulating the resumption and progression of oocyte meiotic maturation before ovulation to further ensure their normal developmental potential [22,23]. In rat oviducts, prostaglandins participate in the regulation of oocyte transport [24]. However, *COX-2* expression pattern varies between different mammals, which suggests that *COX-2* transcript and the encoded protein could have varying functions in different species. 

The oviductal environment and development of the oocyte maturation are influenced by the paracrine activity from the oviductal cells and the cumulus cells released with the cumulus-oocytes complexes (COCs) during ovulation [8]. Growth factors like TGF-β superfamily have been detected in the oviduct of mammalian species [25,26]. Among the member of this family, the growth differentiation factor 9 (GDF-9) and bone morphogenetic protein 15 (BMP-15) regulate a variety of reproductive functions through the activation of several signalling pathways [27,28,29]. In previous studies, we demonstrated the improvement in attaining the final stages of meiosis during in vitro maturation (IVM) of canine cumulus-oocytes complexes (COCs) when recombinant GDF-9 and BMP-15 proteins were added together to the culture media [30]. In addition, GDF-9 and BMP-15 appear to upregulate the levels of *COX-2* transcripts during the in vitro maturation of canine oocytes [21]. Accordingly, GDF-9 in mice has been reported to cause more than 50-fold increase in the *COX-2* expression [31]. These paracrine factors play a crucial role in oocyte development, but little is known about their expression and function in the oviduct. The canine oviducts undergo physiological and hormonal changes according to the ovarian cycle [32]. According to our previous studies, the mRNA levels of *GDF-9* and *BMP-15* in canine follicles and COCs are not expressed equally during the follicular development throughout the ovarian cycle, suggesting a specific regulation and temporal changes in their expression [33,34]; thus, the function and dynamics of gene expression of *GDF-9* and *BMP-15* in the oviducts may vary in the same way due to these changes. Identifying genes associated with developmentally-competent canine oocytes during maturation at the oviduct level would be important since the change in the abundance of these genes during the oestrous cycle may help to understand the final oocyte growth in dogs. Therefore, the aims of this study were to investigate the oestrous cycle-dependent changes of relevant genes such as *PR*, *COX-2*, *BMP-15*, and *GDF-9* in the canine oviduct and also, the possible influence of ovulated oocytes and its cumulus cells in the expression of *BMP-15*, and *GDF-9*.

## 2. Materials and Methods

Animal procedures involved in this study were approved by the Institutional Bioethics of the National Foundation for Scientific and Technological Research, ANID, the Ministry of Sciences and Technology. The University of Chile Ethical Animal Care Committee also approved this study (9 June 2017, number 08-2017 VET-UCH). Written consents were obtained from the owners of the female dogs.

### 2.1. Sample Collection

**Figure 1:**
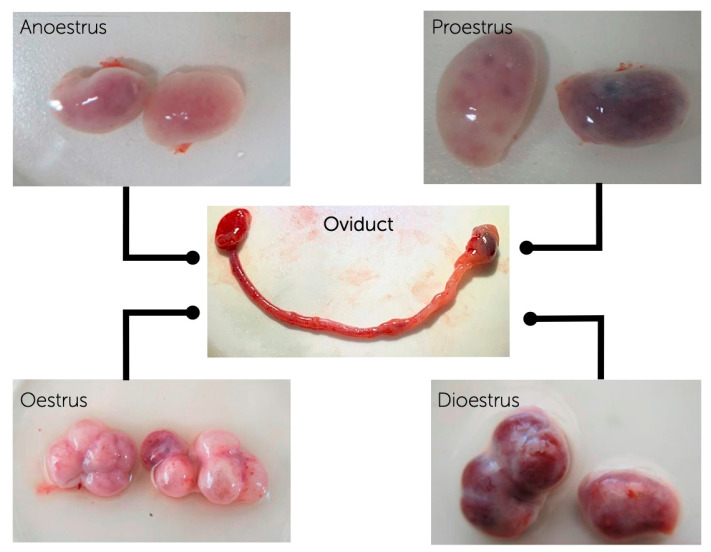
Oviductal samples were evaluated throughout 3 replicates using 3 pools of oviductal cells from oviducts at different phases of the oestrous cycle (anoestrus, proestrus, oestrus, dioestrus) for mRNA, messenger RNA evaluation. Canine ovaries and oviducts at anoestrus, proestrus, oestrus and dioestrus were obtained after routine ovariohysterectomy.

The mixed-breed female dogs with the ages between 1–4 years, at different phases of the oestrous cycle (anoestrus = 9, proestrus = 7, oestrus = 8, and dioestrus = 11) were used for the first part of the experiments. Ovaries, oviducts, and part of the uterine horns were collected and transferred to the laboratory in physiological saline solution (0.9% *w*/*v* NaCl) containing penicillin G (100 IU/mL) and streptomycin sulphate (50 mg/mL) at 37 °C, within 2 h after the routine ovariohysterectomy. The oviducts were classified into the different phases of the oestrous cycle according to the morphology of growing follicles and/or corpus luteum (CL) on the surfaces of the corresponding ovaries as previously described [33] (Figure 1). To confirm the oestrous phase, plasma progesterone levels in blood samples obtained during the surgery were also assessed according to the previous studies [21,34]. In brief, 5 mL of blood samples without anticoagulant were centrifuged at 2500× *g* for 10 min. The supernatants were used in analyses. The progesterone concentration of each blood samples was evaluated in duplicates by an enzyme-linked fluorescence assay (ELFA) on the Mini-Vidas automated analyser (Biomerieux, Marcy l’Etoile, France) [35], using progesterone (P4) canine kits (VIDAS^®^ Progesterone #30409, Biomerieux). The mean coefficients of variation were 3.2 (%) and 5.3 (%) for the intra and inter assays, respectively. The minimal limit of detection was 0.25 ng/mL [21]. Bitches were considered in anoestrus when progesterone values were less than 0.5 ng/mL; proestrus 0.6–1.9 ng/mL; oestrus 2–19 mg/mL; diestrous more than 20 ng/mL [34].

### 2.2. Recovery of the Oviductal Cells

The oviducts were separated from the uterine horns and dissected from the ovarian bursa and trimmed free of surrounding tissues, and then placed into another disposable petri dish (Falcon, Becton Drive Biosciences, Franklin Lakes, NJ, USA) containing phosphate-buffered saline (PBS) (137 mM NaCl, 2.7 mM KCl, 4.3 mM Na_2_HPO_4_, 1.47 mM KH_2_PO_4_, pH 7.4). Under a stereoscopic microscope (Motic SMZ-171, Motic, Vancouver, Canada), the oviductal epithelial cells were collected through gentle pressure with the handle of tweezers on the oviducts. The epithelial cells of each oviduct were resuspended in 1.0 mL of PBS in microfuge tubes and then washed via centrifugation at 700× *g* for 5 min (Eppendorf Centrifuge 5415 D, Eppendorf, Hamburg, Germany). The remaining pellets were transferred separately to a storage reagent, RNAlater^®^ (Invitrogen^TM^ Eugene, OR, USA) to preserve the RNA integrity and then stored in pools according to the oestrous phase (anoestrus, proestrus, oestrus, and dioestrus).

**Figure 2:**
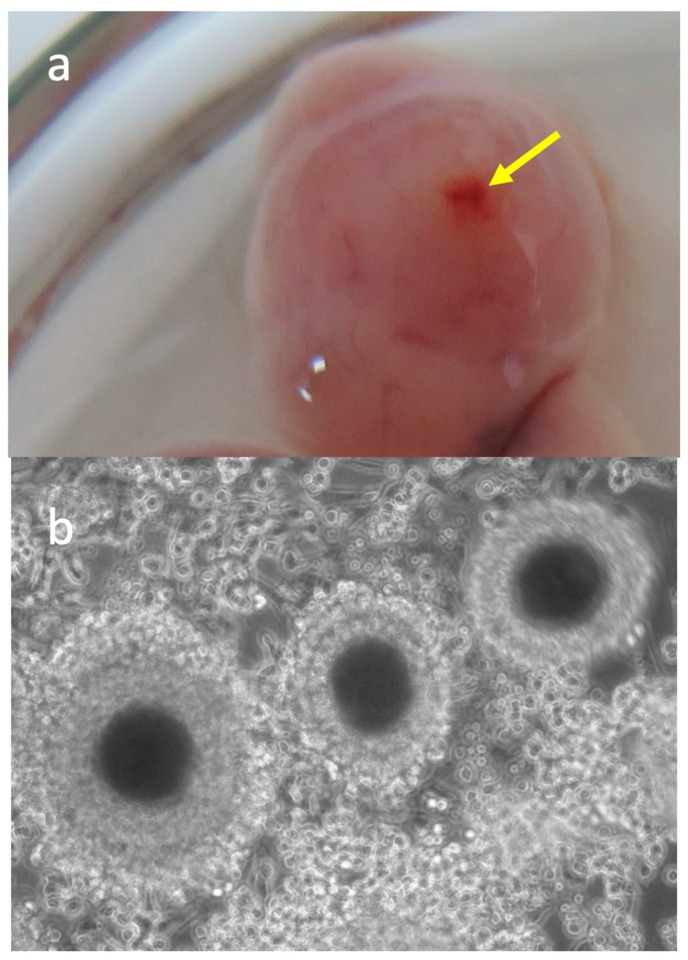
Representative photos of (**a**) canine ovary with ovulated follicle. The arrow shows the ovulation fossa in the top of the follicle; (**b**) ovulated canine oocytes collected from the oviducts (×300).

Considering that GDF-9 and BMP-15 are paracrine factors mainly produced by the oocyte, we proposed in the second part of the study to address the influence of the oocyte and its cumulus cells on the abundance of *GDF-9* and *BMP-15* mRNA in the oviducts. For this, additional oviductal samples from both oviducts of twelve bitches in the oestrus phase were separated into two groups, with ovulated COCs (n = 7) and those from oviducts before ovulation (n = 5), without COCs. The ovulation was assumed to have occurred when the serum progesterone concentration reached 8 ng/mL, and the ovaries showed large antral follicles with a visible ovulatory fossa (Figure 2a). In addition, the presence of ovulated COCs (Figure 2b) was confirmed by observing the oviductal samples under a stereomicroscope (SMZ-171; Motic). All COCs were removed from oviductal cells with a small-bore pipette before processing the oviductal cells for gene expression studies.

The oviductal cells were washed in PBS, processed as described before, and then transferred to the storage reagent, RNAlater^®^ (Invitrogen) at −80 °C for further qPCR analysis.

### 2.3. RNA Extraction and Quantitative Real-Time RT-qPCR Analysis

The real-time RT-qPCR was performed using oviduct cell samples from each collected pool and phase of the oestrous cycle to determine the mRNA levels of *PR*, *COX-2*, *BMP-15*, and *GDF-9*.

The total RNA was extracted from the oviduct epithelial cells using the column affinity purification kit GeneJET^TM^ RNA (Thermo Fisher Scientific TM, Waltham, MA, USA) following the manufacturer’s protocols. The concentration of the RNA was determined with a Qubit^®^ Fluorometer (Invitrogen) using the quantification kit Qubit RNA assay (Invitrogen). Reverse transcription was assessed using the enzyme conjugate SuperScript^TM^ first-strand synthesis system (Invitrogen). The complementary DNA (cDNA) concentration was determined using the quantification kit ssDNA Qubit Assay (Invitrogen). Specific primers (Table 1) were used for reverse transcription (Midland Certified Reagent). The RT-qPCR assays were run in duplicates with 10 ng of complementary DNA (cDNA) in an 18 mL total reaction volume. As negative controls, reactions containing no template reverse and transcriptase were included in each plate. The *β-actin* RNA (*ACTB*) was used as the normalization control gene, according to our previous studies [33]. In brief, we used the Norm Finder algorithm, which generates a stability measure for which a lower value indicates increased stability in gene expression, using samples taken from different groups to allow direct estimation of the variation in expression of different candidate genes. The more suitable gene for normalization was *ACTB*. PCR reactions were assessed using the Maxima SYBR Green/ROX qPCR Master mix Kit (Thermo Fisher Scientific TM, Waltham, MA, USA), according to the manufacturer’s instructions. Amplification was performed using the two steps real-time Eco™ PCR system (Illumina^®^, San Diego, CA, USA). The 2^−∆∆CT^ method was used to transform threshold cycle values (Ct) into normalized relative expression levels of mRNA [36].

### 2.4. Statistical Analysis

The experiments were conducted with a minimum of three independent replicates.

Multiple comparisons of the relative expression levels of *GDF-9*, *BMP-15*, *COX-2*, *PR* mRNAs in oviductal cells between each reproductive phase were analysed by ANOVA. Differences among the means were evaluated using Duncan’s test.

The comparison of *GDF-9* and *BMP-15* gene expression before and after ovulation were assessed using the Student *t*-test.

All analyses were performed using the Info Stat Professional Program, Version 2018; (National University of Córdoba, Argentina). The data were transformed into a normal distribution before applying the analysis.

Pearson’s coefficient correlation analysis was used to test the correlation between plasma progesterone concentration and progesterone receptor (*PR*) gene expression in oviductal cells throughout the oestrous cycle. Differences *P* ≤ 0.05 were considered significant.

## 3. Results

A total of three cell pools were collected from the oviducts in every phase of the oestrous cycle.

The plasma progesterone profiles showed differences according to each oestrous phase in relation to ovarian structures as previously described in other studies [33]. Progesterone values of the donors submitted to ovariohysterectomy were undetectable to 0.4 ng/mL in anoestrus phase, 0.9 to 1.03 ng/mL, in proestrus, 6.20 to 16.02 ng/mL in oestrus and 18.01 to 30.10 ng/mL in dioestrus. These values are within the normal ranges for canines [9,33].

**Figure 3:**
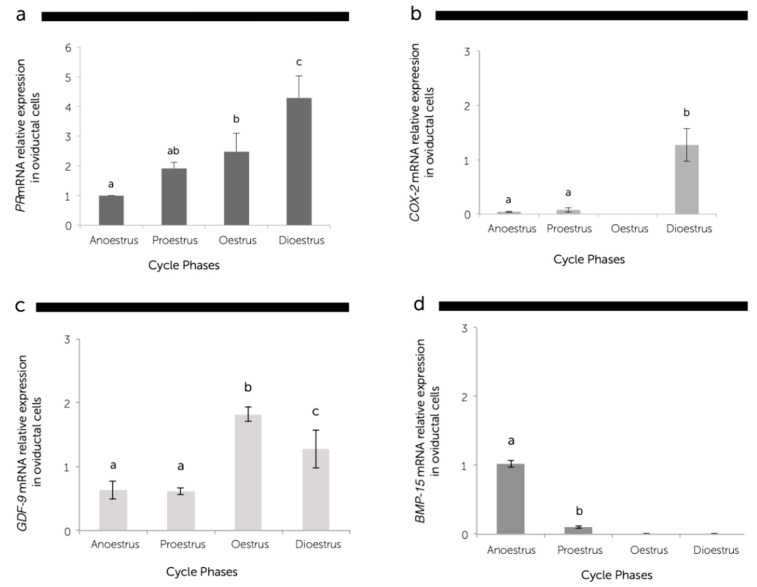
The relative messenger RNA (mRNA) expression of the progesterone receptor (*PR*) (**a**); cyclooxygenase-2 (*COX-2*) (**b**); growth differentiation factor 9 (*GDF-9*) (**c**); bone morphogenetic protein 15 (*BMP-15*) (**d**), standardized with mRNA expression of beta-actin in canine oviductal cells during the anoestrus, proestrus, oestrus, and dioestrus phases. The mRNA levels were expressed in relation to *β-Actin* mRNA as the control or housekeeping gene. Different letters above the bars indicate differences at *P* < 0.05.

The expression of *GDF-9* and *PR* mRNA was detected in the oviducts during the whole oestrous cycle, but the *BMP-15* gene expression was detected only in the anoestrus and proestrus phases, whereas *COX-2* was not detected in oestrus. The abundance of these transcripts was differently expressed in a cycle stage-dependent manner (Figure 3).

**Figure 4:**
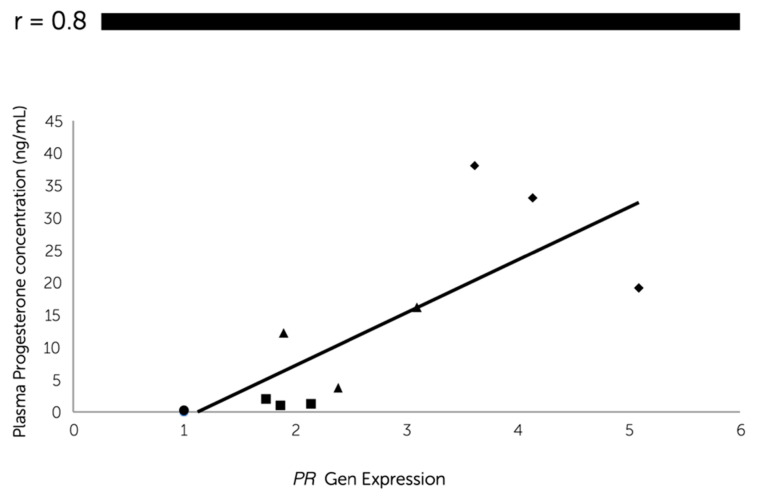
Pearson’s correlation coefficient between the relative expression of the progesterone receptor (*PR*) messenger RNA (mRNA) in canine oviductal cells and the concentration of progesterone in plasma samples obtained in anoestrus (●) (the values in anoestrus were almost the same, thus the points are overlapped); proestrus (■); oestrus (▲) and dioestrus (◆) phases. (*P* < 0.05).

Quantitative real-time RT-PCR revealed a significantly higher gene expression of *PR* in the dioestrus phase than the other phases (Figure 3a). The *PR* mRNA transcript gradually increased (*P* < 0.05) from the anoestrus to dioestrus phase with the plasma progesterone concentration, since a significant positive correlation (r = 0.8) was found between progesterone systemic levels and *PR* mRNA abundance at the oviductal level (Figure 4).

A low level of *COX-2* gene expression was observed in the anoestrus and proestrus phases, and there was almost no expression in the oestrus phase. However, the specific mRNA transcript of *COX-2* was expressed around 10-fold higher (*P* < 0.05) in the dioestrus in comparison to the other phases (Figure 3b).

The *GDF-9* gene expression was observed in the oviducts during all phases of the oestrous cycle; however, the highest (*P* < 0.05) abundance was obtained during the oestrus period, followed by the dioestrus phase (Figure 3c). The expression of *BMP-15* mRNA decreased (*P* < 0.05) from anoestrus to proestrus phases, and the expression of this gene was not observed in the oestrus and dioestrus phases (Figure 3d).

**Figure 5:**
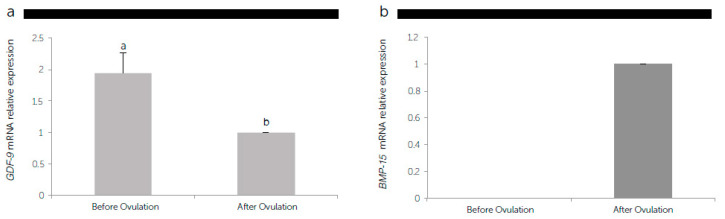
The relative messenger RNA (mRNA) expression of the growth differentiation factor 9 (*GDF-9*) (**a**), and bone morphogenetic protein 15 (*BMP-15*) (**b**) standardized with mRNA expression of *β-actin* mRNA in canine oviductal cells before and after ovulation. Different letters above the bars indicate differences at *P* < 0.05.

Comparing the *GDF-9* and *BMP-15* gene expression pattern in the oviduct samples obtained during oestrus without (before ovulation) or with COCs (after ovulation) (Figure 5), the *GDF-9* mRNA levels decreased (*P* < 0.05) by almost 50% after ovulation under the influence of COCs (Figure 5a). On the contrary, *BMP-15* transcript was expressed only when the oviduct cells were obtained after ovulation with the presence of COCs (Figure 5b).

## 4. Discussion

A better understanding of the gene expression in the oviducts could help us progress in canine reproduction and improve in vitro culture conditions used in reproductive biotechnologies. To the best of our knowledge, this is the first report to study the expression of *PR*, *COX-2*, *BMP-15*, *GDF-9* genes in the canine oviducts throughout the four phases of the oestrous cycle in canines, demonstrating that the level of each gene was related to the cycling ovary, undergoing variations with each oestrous phase. These differences in the local milieu may be involved in the change in oviductal functions throughout the oestrous cycle.

The cyclic changes in *PR* transcript in the oviduct were following the changes observed in the canine progesterone serum samples. They agreed with the reports in other species, where progesterone’s actions have been reflected in the concentrations of its receptor in the target cells [37]. Therefore, a positive correlation between the serum progesterone concentration and the gene expression of *PR* in the oviducts was observed with increasing *PR* transcripts abundance from anoestrus to dioestrus with the highest *PR* expression during the dioestrus phase. However, earlier report localized the PR protein in the canine oviducts throughout the oestrous cycle by immunohistochemical staining, demonstrating the presence of PR in all phases with the intensity of the stain significantly higher during proestrus than in late dioestrus and anoestrus [38]. Transcription and translation are distinct processes with different timings and mechanisms of regulation despite serving a common purpose [39]. The progesterone receptors are dependent on the presence of oestrogen [40]; therefore, it is possible that PR is more preferentially translated during the proestrus phase than during the other phases, because in that period the oestrogen levels are high in bitches [9,21]. However, the highest *PR* gene expression during dioestrus phase found in our study might explain an active transcriptional activity, possibly stimulated by the luteinizing hormone (LH), considering that the expression of PR mRNA is positively regulated by the LH surge [41]. The PR mRNA upregulation increased in the oestrus and dioestrus at the surge of LH previously because this event occurred at the beginning of the oestrus phase in this species [9]. Several pieces of evidence support the hypothesis that progesterone levels are associated with mammalian oocyte maturity [42,43]; this hormone seems to be responsible for the resumption of meiosis in oocytes leading to an increase in the intracellular calcium [44]. Since the canine oocytes are matured after ovulation, outside the follicle environment in the oviducts and the increased gene expression of PR was observed at this level after ovulation, this may suggest that PR might be involved in the creation of the optimal environment for the maturing oocyte directly or indirectly.

Many cellular signalling genes have been identified as being PR-regulated. The most putative PR target identified in the oviduct is the prostaglandins [45]. Prostanoids are produced by the *COX-2* pathway [46]. In the current study, the relative abundance of *COX-2* in the oviduct showed the most remarkable expression during dioestrus, with very low levels in the other phases in contrast with the increasing pattern of *COX-2* expression previously reported in canines at the follicular level during oestrous [21]. The high concentration of progesterone during dioestrus in dogs might be involved in *COX-2* gene expression in the oviductal cells during this phase. The LH surge-induced changes in the expression of *COX-2* and possibly such effects are potentially mediated, at least in part, by the progesterone-induced regulation of the *COX-2* gene [47,48]. In the bovine oviduct in vitro, *COX-2* mRNA expression was stimulated after direct progesterone treatment [19]. Considering this high *PR* and *COX-2* mRNA levels expressed during dioestrus in this study, possibly occurring through prostaglandin synthesis, the early embryo transport is facilitated by the stimulation of oviductal smooth muscles [49] and changing the secretory conditions.

Although the relevance of GDF-9 and BMP-15 proteins in reproductive processes, such as follicular development and oocyte competences, are well-known, in few studies have measured these paracrine factors in the oviductal cells, maybe because both these factors are thought to be expressed only by the oocytes. However, other reports have demonstrated its presence in the oviduct and other tissues [26,50]. *GDF-9* was expressed during the entire oestrous cycle in the canine oviducts; however, the highest relative abundance was observed during the oestrus phase, which is the opposite to the lowest expression reported earlier at a follicular level during the oestrus stage in dogs [33,34], which suggests that the extent of the expression of this gene depends upon the cell type. The importance of this factor in oocyte maturation has been demonstrated in vitro in different species [51,52], including canines [30]. Therefore, *GDF-9* and its encoded protein may implement a similar function in the oviductal tissues to promote the conditions for oocyte maturity in dogs. For this purpose, the existence of a local regulatory mechanism is possible at this level. Considering that the canine oocyte maturation occurs in the oviduct, it is conceivable that these mRNAs present in the oviductal cells may be transferred to the oocytes for their maturation. Furthermore, GDF-9 inhibits the follicle-stimulating hormone (FSH)-induced steroidogenesis while promoting progesterone production [53]. Interestingly, after the ovulation process, the presence of the oocyte decreased *GDF-9* expression in the oviductal cells, compared to the transcript abundance before ovulation. The decreasing *GDF-9* levels in the oviducts after ovulation might be associated with the meiosis resumption, because during this process when the proteins are required, the transcripts are polyadenylated and used for translation, then rapidly degraded.

In contrast to *GDF-9*, the *BMP-15* gene expression was only observed in anoestrus and proestrus. Samples collected at anoestrus and proestrus in dogs have not been exposed to the endogenous LH surge because the physiological proestrus in this species is completed at the moment of the LH surge [9]. The LH surge induces multiple intracellular signalling and second messengers in many reproductive cells [54,55], influencing the gene expression. Relative oocyte abundance of *BMP-15* in mice decreases significantly after human chorionic gonadotrophin (hCG) treatment [56], providing evidence that the preovulatory LH surge leads to regulating this gene. Follicles and the oviducts express similar cell-signalling genes that have the potential to participate in the regulation of development of the oocyte. Therefore, the preovulatory rise in LH that eliminates BMPs, enabling luteinization to progress in the follicle [57], could also affect the oviductal cells. In the same way, the highest expression of BMP factors was detected during the preovulatory stage in bovine, suggesting a possible oestrogen-regulated expression [58].

The high abundance of *BMP-15* observed in anoestrus phase is not clear, as yet the role of BMP-15 in the regulation of oviductal cells has not been defined. However, the high gene expression of *BMP-15* during this phase supports the notion that this gene, like other members of the BMPs system, could play an important role in the regulation of oviduct function during the anoestrus period by the ability to control cell proliferation and cytodifferentiation [30], preparing the oviduct for the next reproductive stages. However, its exact function needs to be further studied.

After ovulation, the presence of COCs increased the levels of *BMP-15*, this indeed suggests that many *BMP-15* transcripts observed after ovulation came from the transcriptional activity of the cumulus cells released with the COCs at ovulation. Furthermore, the oviduct milieu is influenced by the paracrine function of the cumulus and granulosa cells released with the oocyte at ovulation [8]. Direct interactions among cells inducing cellular signalling in the oviductal cells are important regulatory mechanism during the progression of the oestrous cycle [43].

## 5. Conclusions

This study demonstrated the influence of the ovarian cycle on the essential oviductal genes involved in the oocyte maturation in bitches. Moreover, the effect of the ovulated COCs on the gene expression of *GDF-9* and *BMP-15* in the oviduct was noticeable. Therefore, the use of oviductal cells in a defined culture system for in vitro maturation protocols or embryo culture should consider the oviductal stage through the oestrous cycle. Further studies on the effects of the proteins encoded by each of the genes evaluated herein in the canine oviducts can be useful to identify additional information and signalling pathways molecules that act on oocyte and embryonic development.

## Figures and Tables

**Table 1:** Sequences of specific primers and reference gene used in this study for qPCR analysis.

Gene	Sequence 5′-3′	Size (bp)	Reference
*ACTB*	F:ATTGTCATGGACTCTGGGGATG R:TCCTTGATGTCACGCACGAT	191	[33]
*GDF-9*	F: CAGAAGGGAGGTCTGTCTGC R: TGTTGGGGGAAAAGAAAGTG	170	[33]
*BMP-15*	F: CCCTGCCCCTGATTCGGGAG R: CCGCAAAGGATGCCCAAGGAC	82	[33]
*COX-2*	F:TGAGCGGTTATTCCAGACGAGCAG R:CCAACCCCGCAGCCATTTCCTTCT	500	[21]
*PGR*	F:GATGCTATATTTTGCACCTGA R: CTCCTTTTTGCCTCAAGCCA	266	[15]

Abbreviation: qPCR, quantitative real-time polymerase chain reaction; *PGR*, progesterone receptor *COX-2*, cyclooxygenase-2, *GDF-9* growth differentiation factor 9, and *BMP-15,* bone morphogenetic protein 15.

## Data Availability

The data presented in this study are available on request from the corresponding author.
